# Superficial Arterial Variants of the Upper Limb: Clinical Implications of High-Origin Ulnar and Radial Arteries Detected by Ultrasound and Anatomy Study

**DOI:** 10.3390/jfmk10030246

**Published:** 2025-06-27

**Authors:** Maribel Miguel-Pérez, Sara Ortiz-Miguel, Ana Martínez, Juan Carlos Ortiz-Sagristà, Ingrid Möller, Carlo Martinoli, Albert Pérez-Bellmunt

**Affiliations:** 1 Unit of Human Anatomy and Embryology, Department of Pathology and Experimental Therapeutics, Faculty of Medicine and Health Sciences (Bellvitge Campus), University of Barcelona, 08007 Barcelona, Spain; amartineztorres97@gmail.com (A.M.); ingrid.moller@gmail.com (I.M.); 2 Unit of Human Anatomy and Embryology, Department of Surgery and Medical-Surgical Specialities, Faculty of Medicine and Health Sciences (Clinic Campus), University of Barcelona, 08007 Barcelona, Spain; saraormi94@gmail.com; 3 Medicine Department, Faculty of Medicine and Health Science, Universitat Internacional de Catalunya, 08017 Barcelona, Spain; aperez@uic.cat; 4 Actium Functional Anatomy Research Group, 08195 Sant Cugat del Vallès, Spain; 5 Anesthesiology Department, Fundació Puigvert, 08025 Barcelona, Spain; siometge@gmail.com; 6 Cattedra di Radiologia “R”-DICMI, Universita di Genova, 16126 Genova, Italy; mskeletal.radiology@gmail.com

**Keywords:** superficial ulnar artery, radial artery variation, vascular anomalies, ultrasound anatomy, clinical safety, anatomy

## Abstract

**Background:** Arterial variations in the upper limb, although infrequent, carry critical clinical implications. The presence of superficial ulnar and radial arteries, especially when originating from high levels, increases the risk of iatrogenic injury, misdiagnosis, and surgical complications. To confirm and describe, through ultrasound and anatomical dissection, the presence of a high-origin superficial ulnar artery and a superficial radial artery in a cadaver, highlighting their anatomical trajectory and clinical relevance. **Methods:** A cross-sectional ultrasound and anatomical study was conducted on 150 upper limbs from fresh-frozen cadavers. High-frequency ultrasound was used to scan the vasculature from the axilla to the wrist. Subsequently, dissection was performed to confirm sonographic findings. **Results:** One case (0.66%) of concurrent superficial ulnar artery and superficial radial artery was identified in the left arm of a 79-year-old male cadaver. The superficial ulnar artery originated from the axillary artery and coursed superficially along the forearm, anterior to the flexor muscles. The superficial radial artery emerged from the brachial artery and ran subcutaneously in the distal forearm. These arteries remained in close relation to key neural and venous structures, increasing their vulnerability to clinical error. **Conclusions:** The identification of high-origin superficial arteries is essential for clinical practice. Ultrasound serves as a reliable, non-invasive method for detecting such variations preoperatively. Awareness of these anomalies can prevent inadvertent vascular injuries, improve diagnostic accuracy, and inform safer surgical and anesthetic approaches in upper limb interventions.

## 1. Introduction

Classical anatomical texts describe the brachial artery as a direct continuation of the axillary artery, which typically terminates at the anterior aspect of the elbow, medial to the biceps brachii tendon, by dividing into its two main terminal branches: the radial artery and the ulnar artery [1]. The radial artery courses laterally, running in proximity to the sensory division of the radial nerve and beneath the brachioradialis muscle, before reaching the wrist, where it divides into the radial and palmar radial arteries. The UA, on the other hand, travels inferomedially, deep to the median nerve and the superficial flexor muscles of the forearm [1]. It gives rise to the common interosseous artery (CIA), which subsequently divides into anterior and posterior interosseous branches. The radial artery then continues its path adjacent to the ulnar nerve and deep to the flexor carpi ulnaris muscle until it pierces the antebrachial fascia proximal to the flexor retinaculum at the wrist [1]. Within the hand, the radial artery contributes to the formation of both the superficial and deep palmar arches via the deep palmar branch, along with the superficial palmar branch of the radial artery and the radial artery itself, respectively [2].

Despite this well-established anatomical pattern, numerous studies have documented significant arterial variations in the upper limb vasculature [2,3]. Among these, the superficial radial artery, which arises directly from the brachial artery, has been observed with a prevalence of 14.26–15% [4]. Another notable variant is the superficial ulnar artery, which may originate from either the axillary or brachial artery and follow a superficial trajectory over the forearm flexor muscles [4]. The prevalence of the superficial ulnar artery in the general population ranges from 0.7% to 9.4%, with bilateral occurrence being more common [1,5].

These vascular anomalies are not merely of academic interest; they carry significant clinical implications. Superficial arteries are particularly susceptible to traumatic injury and iatrogenic complications during surgical or percutaneous procedures involving the forearm [3]. Furthermore, due to their atypical superficial location, these arteries can be misidentified as veins, leading to inadvertent intra-arterial injections with potentially severe consequences [6]. Consequently, early and accurate identification of such variations is critical in minimizing procedural risk and improving patient safety. Imaging techniques such as Doppler ultrasound and contrast-enhanced studies have proven valuable in detecting these anatomical variants preoperatively or during diagnostic evaluation [7].

The objective of this study is to emphasize the clinical relevance of performing a thorough Doppler ultrasound examination of the upper limbs prior to invasive procedures. Specifically, we aim to assess the prevalence and detectability of high-origin superficial ulnar and radial arteries (superficial ulnar artery and superficial radial artery, respectively). As it has been suggested in previous research about vascular variations, these findings highlight the importance of understanding and identifying such variations to enhance surgical accuracy and patient safety [8].

## 2. Materials and Methods

### 2.1. Study Design and Samples

A cross-sectional ultrasound and anatomical study was conducted. The study obtained approval from the Local Ethics Committee of the Universitat de Barcelona (IRB00003099). All participants in this study signed a written consent to donate their bodies for research. This observational study involved the ultrasound and anatomy of 150 upper limbs from fresh frozen cadavers belonging to 68 males and 82 females, 78 lefts and 72 rights. The age range was 50–93 years.

All limbs had been cryopreserved at −20 °C in the dissection room. The body donor program of the Faculty of Medicine and Health Sciences (Bellvitge Campus) provided all the specimens. None of the anatomical samples had evidence of traumatic injury or surgical scars. Samples that presented interventions, prostheses, or surgical material were excluded.

### 2.2. Ultrasound Study

For the ultrasound study, we used a LOGIQ P6 ultrasound system (GE Ultrasound Korea, Ltd., Seongnam, Republic of Korea) with a 6–15 MHz frequency range.

The ultrasound study began with the upper limb in the anatomical position, from the anterior and superior side of the arm, continuing down to the hand. A special study of all anatomic and anomalous structures was performed.

### 2.3. Anatomical Study

After the ultrasound study, a careful dissection of the upper limb was performed following the classical pattern. It began on the anterior surface of the upper limb with a longitudinal incision and three horizontal lines at the level of the glenohumeral joint, elbow, and wrist. The dissection then continued in planes: the skin, subcutaneous tissue, and superficial fascia were discarded, leaving the deep fascia and other anatomical structures exposed.

## 3. Results

An arterial variation was identified only on the left upper limb (from 150 upper limbs) of a 79-year-old male cadaver. No vascular anomalies were found in the contralateral limb or in any of the other specimens examined. Therefore, the incidence of this variation among the dissected specimens was 0.66%.

### 3.1. Ultrasound Study

Ultrasound evaluation of the upper limbs revealed the normal musculoskeletal and vascular structures in most cases. However, in one individual (a 79-year-old male), a notable arterial variation was detected in the left upper limb. On ultrasound imaging of the anterior and medial aspect of the axilla, a round, hypoechoic structure corresponding to the axillary artery was visualized, surrounded by the terminal branches of the brachial plexus.

**Figure 1:**
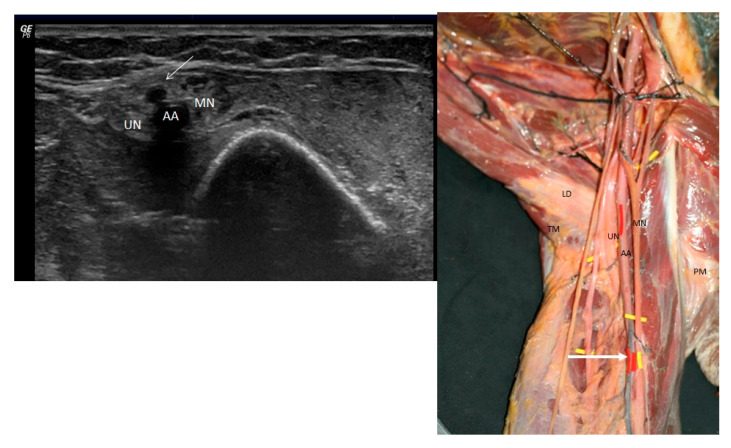
(**Left**) Ultrasound view of the axillary artery (AA) and the superficial ulnar artery (SUA) marked with a white arrow in relation to the median nerve (MN) and the ulnar nerve (UN). (**Right**) Dissection of the ulnar nerve (UN) and median nerve (MN) in relation to the axillary artery branches of the brachial plexus and the superficial ulnar artery (white arrow). Pectoral mayor (PM),latissimus dorsi (LD) and teres major muscle (TM).

**Figure 2:**
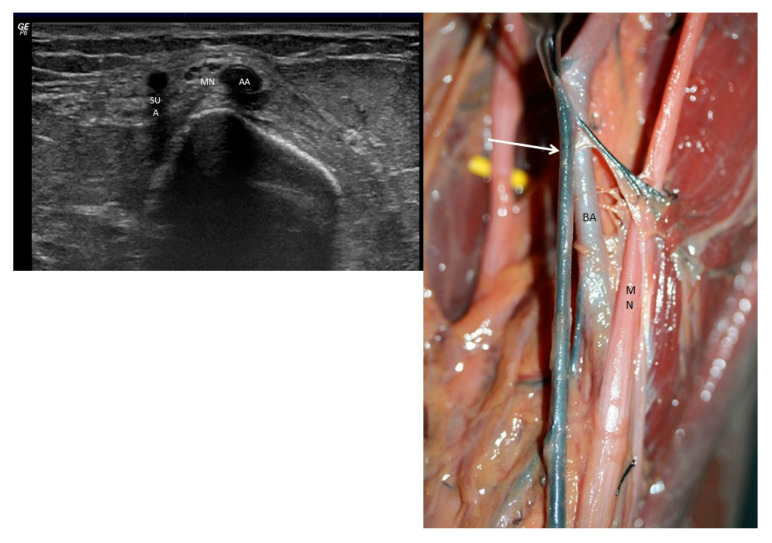
(**Left**) Ultrasound view in the elbow of the superficial ulnar artery (SUA) in a medial position from the median nerve (MN) and axillary artery (AA). (**Right**) Anatomic view of the median nerve (MN) that goes between the superficial ulnar nerve (white arrow) and brachial artery (BA).

In the proximal third of the arm, a smaller-caliber, hypoechoic vessel was observed emerging from the axillary artery and coursing subcutaneously. This vessel was identified as a superficial ulnar artery (Figure 1 left). As the artery descended into the mid-arm, the superficial ulnar artery and the brachial artery (BA) diverged, with the median nerve interposed between them (Figure 2 left). The superficial ulnar artery remained superficial throughout its course, lying just beneath the hypoechogenic skin and subcutaneous tissue.

**Figure 3:**
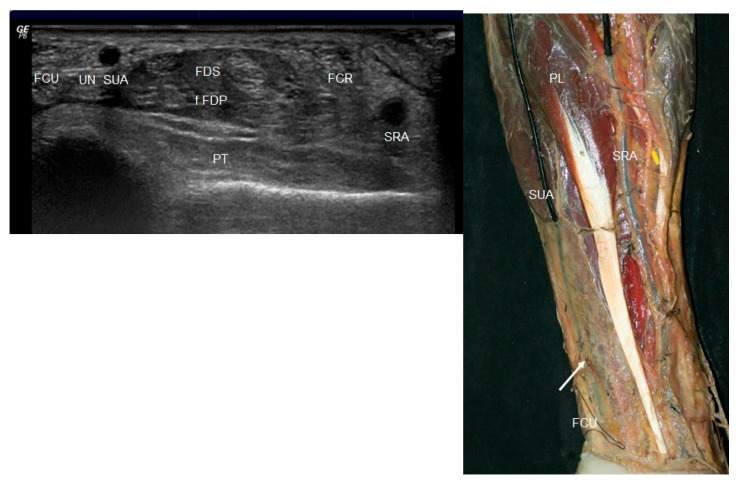
(**Left**) Distal ultrasound view of the forearm with the superficial ulnar artery (SUA) and the superficial radial artery (SRA) in relation to the flexor carpi ulnaris (FCU) and ulnar nerve (UN) flexor carpi radialis (FCR) and pronator quadratus muscle (PT). Flexor digitorum superficialis (FDS) and flexor digitorum profundus (FDP). (**Right**) Dissection of the anterior side of the forearm it could see the palmaris longus (PL), the flexor carpi ulnaris (FCU) and the superficial ulnar artery (SUA) and the superficial radial artery (SAR) under the forearm fascia was not dissected in the inferior third of the forearm.

At the level of the forearm, the superficial ulnar artery was visualized superficial to the flexor digitorum superficialis muscle, situated lateral to the ulnar nerve and the flexor carpi ulnaris. Meanwhile, the brachial artery continued its course and gave rise to a radial artery (RA), which also followed a superficial trajectory as a superficial radial artery, extending into the distal third of the forearm (Figure 3 left).

No additional vascular anomalies were observed in the remaining upper limbs on ultrasound examination; all other vascular structures conformed to the standard anatomical pattern.

### 3.2. Anatomical Study

Gross anatomy studied by dissection procedure confirmed the findings obtained through ultrasonography. In the same 79-year-old male cadaver, once the anterior shoulder muscles were carefully removed, the axillary artery was identified in its expected position, encircled by the terminal branches of the brachial plexus. However, an atypical arterial branch (corresponding to the superficial ulnar artery) originated from the axillary artery in the proximal third of the arm (Figure 1 right). This origin was located just distal to the convergence of the two roots of the median nerve and superior to the inferior margins of the teres major and pectoralis major muscles.

**Figure 4:**
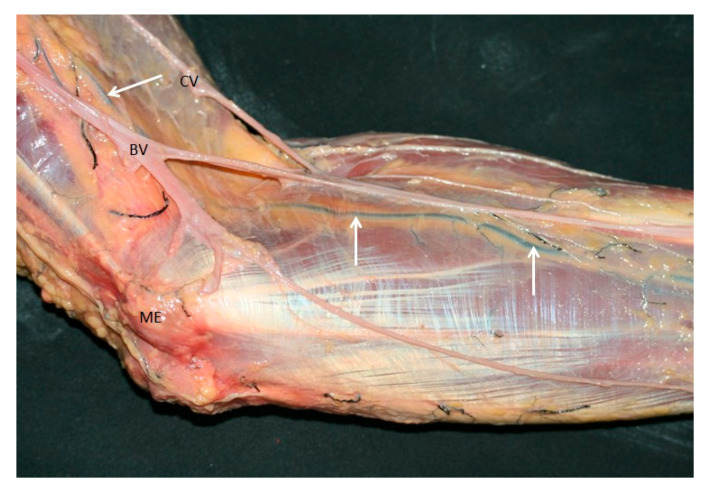
Anatomic vision of the superficial ulnar artery (white arrows) under the fascia of the forearm, between the cephalic (CV) and basilic vein (BV) in the elbow. Medial epicondyle (ME).

The superficial ulnar artery emerged as a superficially positioned artery, lying immediately beneath the brachial fascia and traveling between the cephalic and basilic veins, a relationship maintained even as it entered the forearm (Figure 4). The axillary artery continued its typical path distally as the brachial artery, coursing lateral to and in proximity with the median nerve. In contrast, the superficial ulnar artery—of smaller caliber—descended medially (Figure 2 right).

**Figure 5:**
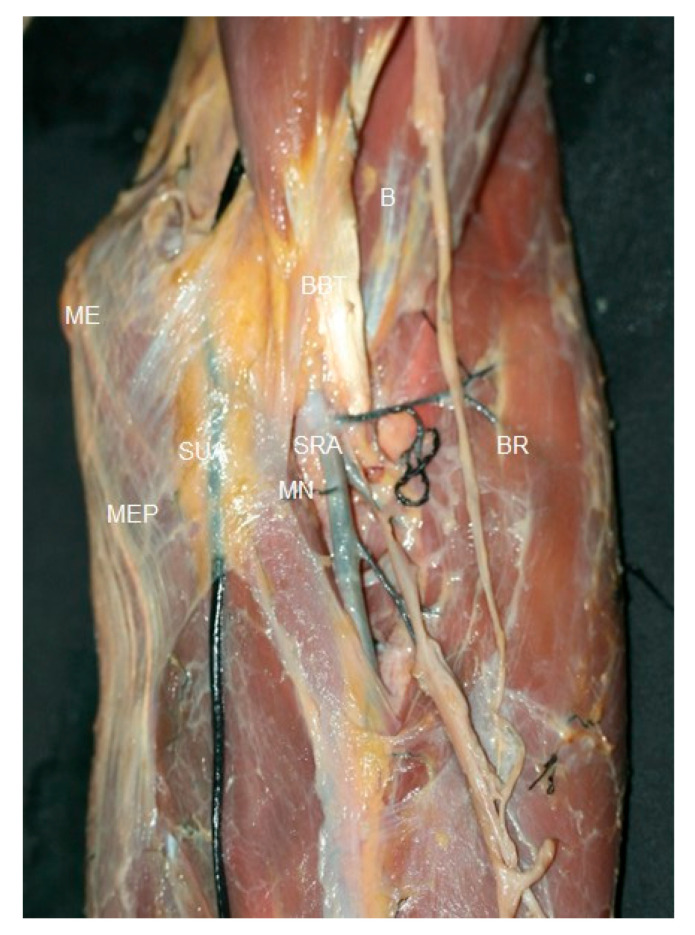
Dissection at the elbow, the superficial ulnar artery (SUA) and superficial radial artery (SRA)covered by the bicipital aponeurosis. Brachial artery (BA) and median nerve (MN) crossed the cubital fossa superficially, medial to the biceps brachii tendon (BBT). Bracial muscle (B), medial epicopndyle (ME), medial epicondyle proximal attachment muscles (MEP), and brachioradialis muscle (BR).

**Figure 6:**
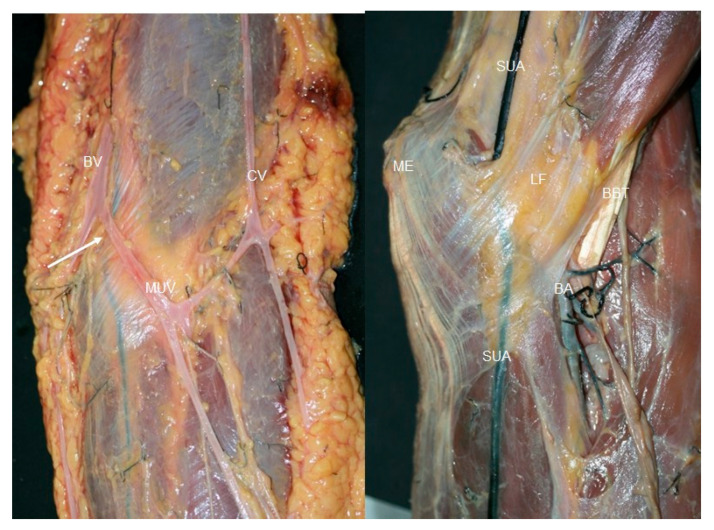
(**Left**) The anatomic dissection shows that before the basilic vein (BV), cephalic vein (CV) superficial ulnar artery (SUV),and white arrow crossed the elbow, it was crossed by the median ulnar vein (MUV), (**right**) and then ran deep to the bicipital aponeurosis (BA) surrounded by adipose tissue. Other structures that can be observed: superficial ulnar artery (SUA), medial epicondyle (ME), brachial artery (BA) and fascia of acertus fibrosus (LF).

Both the brachial artery and the superficial ulnar artery, accompanied by the median nerve, traversed the cubital fossa on a superficial plane, medial to the tendon of the biceps brachii muscle (Figure 5). Throughout its path in the arm, the superficial ulnar artery remained superficial, protected only by the brachial fascia. Just prior to crossing the elbow, it was overlain by the median cubital vein. Subsequently, it passed deep to the bicipital aponeurosis, where it was enveloped in adipose tissue (Figure 6). From there, the superficial ulnar artery descended distally and slightly medially along the forearm, running superficial to the flexor musculature but deep to the superficial venous system. It reached the distal third of the forearm, continuing lateral to the flexor carpi ulnaris and in close relation to the ulnar nerve (Figure 3 right). At the level of the wrist, it passed anterior to the flexor retinaculum, where it bifurcated into its terminal branches.

**Figure 7:**
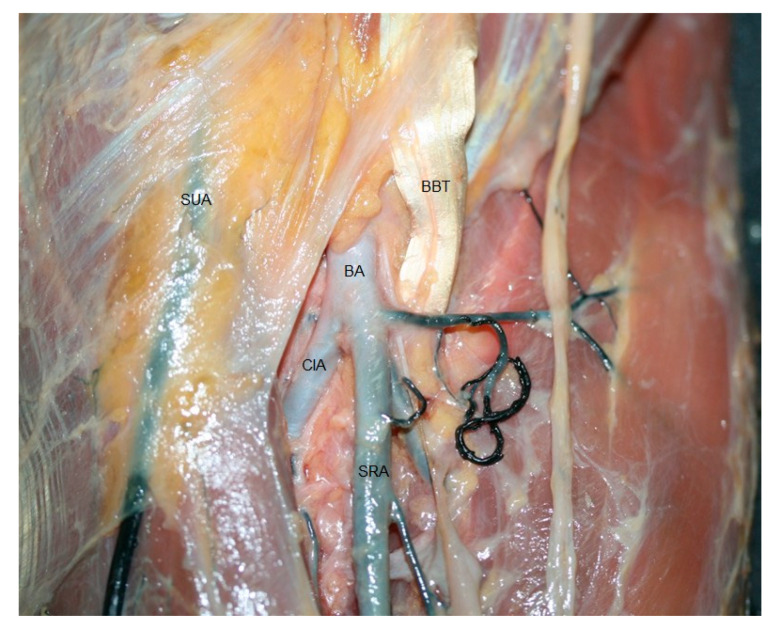
Dissection view of the brachial artery (BA) after crossing the elbow with the superficial ulnar artery (SUA). It gave rise to the superficial radial artery (SRA) and common interosseous arteries (CIA). Other structures that can be observed: biceps brachial tendon (BBT).

In the meantime, the brachial artery (after crossing the elbow together with the superficial ulnar artery) gave rise to the radial artery and the common interosseous artery (CIA). The CIA then divided into medial and lateral branches (Figure 7). In the forearm, the radial artery followed a superficial path like the superficial ulnar artery, traveling medial to the brachioradialis muscle and continuing toward the wrist, where it conformed to the typical anatomical pattern.

The configurations of both the superficial and deep palmar arches were found to be within normal anatomical limits.

## 4. Discussion

To our knowledge, there are no studies that describe using ultrasound and subsequent anatomical study to show the unilateral presence of several variations of a superficial ulnar artery coming from the axillary artery and a superficial radial artery. Injection of the subclavian artery, prior to the study, helped visualize this structure as an anomalous structure in the anteromedial forearm. Careful dissection in planes allowed confirmation of the existence of the superficial radial artery and superficial ulnar artery.

Although upper limb vascular variations are very common [2], the presence of a radial and ulnar artery of high origin is considered a rare anatomical variation with clinical significance [9]. The superficial ulnar artery is a more common anomaly than the superficial radial artery. The superficial ulnar artery is described as having a prevalence of 0.17% to 3.75% [2,9], and our findings of 0.66% prevalence were in line with this. The superficial radial artery, in contrast, is present in <0.2% [2].

Extensive studies of variations of arterial pattern in the upper limb have been reported [1,2]. They have described a high origin of the ulnar artery from the axillary artery [2,6,10] as we found. An even rarer variation [10], from the brachial artery [1] or superficial brachial artery, has been termed the “superficial ulnar artery” [1]. However, some classical authors already described in their anatomical books and on gross dissection the presence of a superficial ulnar artery as a vascular pattern variation. They even specified that the artery separates higher (superior third of the arm), that it is more superficial, and that it has a smaller diameter than the radial artery, as we observed. Also, the superficial radial artery has been described [3] as arising from the division of the brachial artery at the elbow [3], as we found. In the majority of cases, however, the authors describe only one variation [3,11,12]. In our study we observed the superficial ulnar artery arising from the axillary artery, running superficial to the median nerve, under the brachial fascia in the arm, and passing deep into the bicipital aponeurosis along with the brachial artery. It descended on the cubital side of the forearm and beneath the antebrachial fascia along with the superficial radial artery, which was located in the midline of the forearm.

Usually, the CIA, anterior ulnar recurrent artery, and posterior ulnar recurrent artery usually arise from the ulnar artery after it has crossed the cubital fossa, but in this study, the CIA comes from the distal division of the brachial artery after it has crossed the cubital fossa [13,14], unlike other variations that come from the radial artery [1,15].

The CIA may arise from the axillary artery or brachial artery [14]. A high origin of the radial artery and CIA has been reported [7], and even, a case of CIA originating from the brachial artery has been observed [14]. Some authors describe only the bifurcation of the brachial artery into the common radial and interosseous trunk and the superficial course of the ulnar artery [16]. However, this study shows another variation with the superficial radial artery.

The association of absent or inverse palmaris longus muscle with the superficial ulnar artery has been mentioned in the literature [17,18]. In the present study, although the palmaris longus was absent on both sides, the superficial ulnar artery and superficial radial artery were observed on the left side only, as in other studies [16], although the association between the absence of the palmaris longus muscle and the superficial ulnar artery could be purely a coincidental finding.

It may be possible to identify this vascular variation with palpation, however, some authors suggests that the absence of pulsation is an unreliable sign, perhaps in the case of partial occlusion of arterial flow by an applied tourniquet [12]. For this reason, a quick ultrasound assessment can alert about its presence. As we have previously mentioned, ultrasound is a very useful tool to discover this variation. In this study, the prior injection of latex in the artery helped us to recognise it, because we can not use Doppler as other authors do to prove the presence of the superficial ulnar artery [7]. Like them, we saw that the artery could be followed proximally, but they describethe superficial ulnar artery joining the brachial artery just above the elbow skin crease, whereas we observed it coming from a high division of the axillary artery. When we followed this vessel distally, it coursed along the ulnar aspect of the forearm to the wrist, just under the superficial or adipose tissue.

The key finding of this study is that it provides knowledge of the superficial radial artery and superficial ulnar artery, which is clinically relevant for several reasons [5,19]. Multiple clinicians could be involved in its injury. A nurse or anaesthetist looking for peripheral venous access may mistake it for a superficial vein or even a venous variation if the superficial ulnar artery runs under the median cubital vein [9]. Identifying a superficial blood vessel as an artery or vein is not always easy [12], so there is a risk of potential intra-arterial injection, secondary gangrene, and even amputation [20]. A radiologist could misinterpret it as an incomplete angiographic image [21]. Orthopedic surgeons also must avoid damaging it in cases of trauma or in surgical interventions to the anterior side of the upper limb, because it is less protected for a large part of its course [3]. Other surgical specialities should be aware of these kinds of vascular variants. For example, surgeons who create AV access for dyalysis or in reconstructive plastic surgery when they look for large pedicles for raising flaps for local reconstructive surgeries in the axilla, elbow, wrist, or hand region [5].

## 5. Conclusions

The presence of superficial arterial variations such as the superficial ulnar artery and superficial radial artery, particularly when they originate at a high level from the axillary or brachial artery, represents a clinically significant anomaly. These vessels, due to their superficial trajectory and close anatomical relationship with structures commonly accessed in medical procedures, are prone to iatrogenic injury. Misidentification of superficial veins can lead to accidental intra-arterial injections, posing risks of tissue ischemia, necrosis, and even limb loss. Furthermore, their unanticipated presence may complicate surgical interventions in the upper limb, such as orthopedic reconstructions, vascular grafts, or flap harvesting in reconstructive surgery.

The present findings underscore the value of routine preoperative ultrasound assessments of the upper extremities, particularly in patients scheduled for vascular access, regional anesthesia, or surgical interventions in the axillary, cubital, or forearm regions. Early detection of such vascular variants through non-invasive imaging could markedly reduce complications and guide more precise surgical planning.

Although rare, these variations warrant increased awareness across multiple clinical disciplines, including anesthesiology, surgery, interventional radiology, and nursing. By integrating anatomical knowledge with accessible imaging modalities, healthcare professionals can enhance patient safety and surgical outcomes.

## Data Availability

The data presented in this study are available on request from the corresponding authors.

## Abbreviations

The following abbreviations are used in this manuscript:

CIA	Common interosseous artery
US	Ultrasound
SRA	Superficial radial artery
SUA	Superficial ulnar artery
